# Students Participating as Ambassadors for Research in Kentucky (SPARK): A health equity undergraduate research training program

**DOI:** 10.1017/cts.2024.688

**Published:** 2024-12-26

**Authors:** Ariel A. Arthur, Rebecca L. deLacerda Allen, Fidelis Y. Sesenu, Maxwell A. Groznik, Carrie B. Oser, Justin X. Moore, Jessica R. Thompson, Alexis N. James, Madeline N. Dunfee, Philip A. Kern, Nancy E. Schoenberg

**Affiliations:** 1 Center for Health Equity Transformation, University of Kentucky, Lexington, Kentucky, USA; 2 Department of Behavioral Science, University of Kentucky, Lexington, Kentucky, USA; 3 Center for Clinical and Translational Science, University of Kentucky, Lexington, Kentucky, USA; 4 Department of Communication, University of Kentucky, Lexington, Kentucky, USA; 5 Department of Sociology, University of Kentucky, Lexington, Kentucky, USA; 6 Markey Cancer Center, University of Kentucky, Lexington, Kentucky, USA; 7 Department of Internal Medicine, University of Kentucky, Lexington, Kentucky, USA; 8 Massachusetts General Hospital Institute of Health Professions, Boston, Massachusetts, USA; 9 Department of Epidemiology and Department of Biostatistics, University of Kentucky, Lexington, Kentucky, USA

**Keywords:** Underrepresented undergraduates, health equity, mentoring, research training, professional development

## Abstract

The Students Participating as Ambassadors for Research in Kentucky (SPARK) program provides novel health equity research training and targeted mentorship for undergraduates, particularly those from groups underrepresented in the biomedical and behavioral research and workforce. SPARK aims to address inadequate diversity in the medical and scientific research fields by providing comprehensive research mentorship and skill-building. Unlike most existing research training programs that are brief, focus on laboratory research, or are limited to graduate students and junior faculty, SPARK delivers a 16-month intensive behavioral and population health science training, equipping students with needed tools to conceptualize, plan, execute, and analyze their own health equity research study. Trainees complete didactic coursework on health equity, study design and proposal development, data analysis, and ethics. Students receive a stipend and research expenses, and multiple mentors guide them in creating original research projects for which they serve as Principal Investigator. Students disseminate their findings annually at an academic research conference as a capstone. Evaluation data from the first three cohorts suggest SPARK has been pivotal in preparing students for graduate studies and research careers in health equity and behavioral and population health sciences, providing strong support for further investments in similar undergraduate research training models.

## Introduction

People from historically marginalized and minoritized groups continue to be inadequately represented in biomedical and behavioral research and training programs and the workforce [1,2]. Individuals from underrepresented backgrounds often encounter obstacles that hinder their long-term research and workforce success, including systemic inequalities in access to resources and opportunities, lack of mentorship and support networks, biases in recruitment and promotion processes, and limited representation in leadership positions. Financial constraints, family responsibilities, and cultural expectations may further impede their advancement [2]. The greater likelihood of focusing on health disparities research may place such researchers at a disadvantage due to lower rates of funding in this field [3].

Despite these challenges, promoting an inclusive research workforce is critical to improving discovery. For example, research teams with investigators from diverse backgrounds are cited more frequently [4]. Accordingly, the National Institutes of Health (NIH) has identified the diversity of the scientific workforce as a priority, appointing its first Chief Officer for Scientific Workforce Diversity in 2014, and launching the 21^st^ Century Scholars Program in 2021 designed to promote recruitment, retention, and leadership development among diverse staff members. These efforts underline the growing importance of mentorship as a strategy to diversify the research workforce [5,6].

One approach to rectifying the deficiencies in our research workforce involves early introduction to research and training in health equity. While many excellent research training programs include individuals from underrepresented groups[7–11]; many limit their focus to graduate students and junior faculty, laboratory-based research[12,13] and STEM fields, or only provide brief training [14]. To better prepare a diverse array of students to pursue health equity careers, faculty within the Center for Health Equity Transformation (CHET), a health equity research center, and the Center for Clinical and Translational Science at the University of Kentucky collaborated to develop a research training program. In this paper, we describe the Students Participating as Ambassadors for Research in Kentucky (SPARK) program presenting initial findings from 2019 to 2021.

## Methods

### Program eligibility, recruitment, and selection process

While students from all backgrounds are encouraged to apply to SPARK, given our project goal, we prioritize applications of students from traditionally underrepresented groups or disadvantaged backgrounds as defined by the NIH [15]. To be eligible, applicants must: 1) be enrolled at one of two large public universities (one of which is a historically Black university located 30 miles from the primary institution) with at least two academic years remaining until graduation, 2) have a GPA of 2.8 or higher, and 3) be interested in developing health equity research knowledge. Recruitment strategies include emails, flyers, and posters that are shared with several units and partners at both universities and on the websites and social media pages of participating institutions. Potential applicants also learn about the SPARK program through presentations at undergraduate research events, classroom visits, and webinars. Applicants must provide a cover letter explaining what has informed their understanding of health equity and how they hope to advance health equity professionally, their resume, three professional references, and a copy of their transcript. Professional references may include former employers, members of civil or religious organizations, or faculty; since applications are assessed on a range of salient qualities, the specific title or role of the referee is less important than their knowledge of the student’s capacities. Accepting a broad array of referees also ensures a more equitable decision-making process [16].

Once completed applications are received, SPARK’s Executive Committee, which consists of the program director, CHET faculty mentors, graduate research assistants (GRAs), and the program administrator, reviews and evaluates the applications based on students’ prior research, leadership experience, academic achievement, and connection to health equity as demonstrated in their cover letter. Finalists are invited for a panel interview, the Executive Committee selects the top six individuals, and applicants are notified of program admission.

Applications to the SPARK program have increased significantly since its inception, from three in the 2019 application cycle to 22 in 2023. To accommodate this demand and with increased funding, the program expanded from three to its current six SPARK students, known as SPARKlers.

### SPARK structure and leadership

The SPARK program includes a faculty training director, administrative support from a manager and program coordinator, and multilevel mentorship support.

### Program components

#### Overview

The SPARK program is conducted during three academic semesters during which SPARKlers receive training in health equity, research design, and human subjects protection. Trainees meet weekly with research and training mentors, develop their research project, submit an institutional review board (IRB) document, and, during the summer, SPARKlers’ projects implement a mentored health equity research project (Table 1), eventually undertaking data analysis and presenting their findings at the annual CCTS conference.

**Table 1.** Students Participating as Ambassadors for Research in Kentucky (SPARK) program components and timeline

	Spring Year 1	Summer Year 1	Fall Year 1	Spring Year 2
**Didactic Learning**				
*Health Equity 101*	X			
Developing Shared Language	X			
Kentucky Health Inequities	X			
Health Inequity Root Causes	X			
Origins of Community Advocacy and Mistrust	X			
Principles of Equitable Community Research	X			
Research Ethics	X			
*Research 101*	X			
Fundamentals of Research	X			
IRB^††^ Workshop	X			
Developing Research Questions & Study Design	X			
Data Analysis & Management	X			
Research Budget Development	X			
Dissemination of Findings	X			
**Research Project**				
Research topic selection & mentor identification	X			
Study design & budget development	X			
Institutional Review Board (IRB)approval		X		
Data collection & management		X		
Data analysis			X	X
Project write up and manuscript development			X	X
Presentation of findings				X
**Professional Development and Enrichment**				
Guest enrichment seminars		X	X	
Experiential visits	X	X	X	
Health equity book club	X	X	X	X
**Mentorship**				
Research Mentor		X	X	X
Center for Health Equity Transformation (CHET) Faculty Mentor	X	X	X	X
CHET GRA	X	X	X	X

#### Didactic training

Students receive didactic training provided by CHET faculty mentors, GRAs, and guest speakers. This training includes an introduction to research ethics and health equity (Health Equity 101) and an introduction to research including study design and data analysis (Research 101). Students learn about the principles of health equity including foundational language and concepts, and the role of equity-based research in addressing health inequities and disparities. The research course provides instruction on human subjects protection (enabling them to submit an application to the IRB), including the history and importance of ethical research conduct, research processes and protocols, data analysis and management, budget development, and dissemination of findings. Students complete the research training component of the program prior to the implementation of their summer research projects. Responding to evaluations provided by SPARKlers, the didactic trainings have expanded to include the full first semester of the program.

#### Research project development and mentorship

SPARKlers select a research topic based on their personal and professional interests, usually aligning with one of the six health-related research priority areas overseen by the UK’s Office of the Vice President for Research. These priority areas (cancer, substance use, cardiovascular disease, diabetes and obesity, neuroscience, and equity) are both significant health disparities in the state[17] and pertinent to students’ lived experiences. SPARK projects focus on a variety of populations experiencing health inequities and use quantitative, qualitative, or mixed-method approaches. Topics have ranged from health assessments among rural communities to chronic illness in racially-minoritized populations (Table 2). Through classroom discussions and formative assignments, CHET faculty mentors and GRAs guide students through the process of developing research questions related to their topic.

**Table 2.** Students Participating as Ambassadors for Research in Kentucky (SPARK) research project topics and associated methods

*Cohorts*	Project topic summaries					
*Cohort 1*	Blood donation motivators and barriers – Survey	Recovery and reintegration of survivors of sex trafficking – Interviews	Physical activity and rural health – Survey			
*Cohort 2*	Patterns of substance use – Quantitative Secondary Data Analysis	Mental health among the incarcerated – Quantitative Secondary Data Analysis	Adult education and childhood developmental disorders – Quantitative Secondary Data Analysis			
*Cohort 3*	Colon cancer knowledge and risk patterns among African immigrants – Survey	Nutrition patterns in rural Kentucky – Interviews	Black maternal health – Interviews	Disability care access – Survey	Lung cancer screening in Appalachian Kentucky – Interview	
*Cohort 4*	Hearing loss comorbidities – Interviews	Environmental contributors to lung cancer risks – Survey	Nutrition and diabetes in rural Kentucky – Survey	Colon Cancer – Survey	Uterine Fibroids – Mixed method – survey and interview	HIV/AIDS –Interview

#### Mentorship selection process

When a student has been admitted into the SPARK program the Executive Committee helps to identify and connect with appropriate faculty members at the University of Kentucky with relevant research expertise. Additionally, the Committee prioritizes mentorship appointments for those faculty members who have experience with mentees, particularly with undergraduate students or those with limited research experiences and students from traditionally underrepresented or disadvantaged backgrounds. All research mentors are interviewed and screened and undergo two orientation sessions, one with other mentors and one with their SPARKler. While not required, research mentors are provided with resources and recommendations for formal mentorship training. In the future, such training will be required.

Safeguards in the system enhance the likelihood of a productive and successful match. In addition to being carefully screened, each research mentor is reviewed and assessed by the Executive Committee twice a year. The basis for this assessment derives, in part, from the weekly touch base meetings between the Graduate Research Assistant and the SPARKler. Additionally, research mentors are assessed on the ability of their mentee to adhere to timelines (e.g., submission of IRB document, preparation of meeting abstract). If research mentors do not meet expectations, SPARK faculty hold a discussion and remediation plan. Thus far, no research mentors have been terminated from the program, though all research mentors are made aware that in the event that inappropriate, unethical, or otherwise problematic behaviors occur, the mentorship arrangement will be terminated.

Research mentors, who receive a stipend for their participation, are expected to meet twice per month with their SPARKler. During these meetings, SPARKlers and research mentors refine their research questions and develop a project that involves community-engaged research in the student’s home community. While students are not required to be Kentucky residents, 5 out of the 11 students in Cohorts 1–3 were from Kentucky. Each student accepted into the program completes community-based research in the location that they consider home. CHET faculty mentors and CHET GRAs assist students with study recruitment when faculty mentors are unfamiliar with the students’ home communities. Once the research proposal is completed, the student and research mentor develop, submit, and revise an IRB application which is a major milestone of the program.

#### Professional development and enrichment activities

Beginning in the first semester, students participate in three categories of professional development and enrichment activities. The first category includes at least three in-person and virtual professional development sessions on mentorship, academic belonging, leadership development, and physician-scientist careers. SPARKlers engage with guest speakers and benefit both from the content of each session and professional network expansion. The second category involves day-long site visits to both urban and rural areas to experience “health equity in action.” These experiential components include field site visits to ongoing NIH-funded community-engaged research projects or practice-based organizations. For example, during a recent urban site visit, SPARKlers observed two statewide Medicaid managed care organizations and public health practitioners working in a local health department-based equity center. The third category of professional development involves a book club where SPARKlers select the specific book and come together to discuss it. The book club consistently is mentioned as a program highlight. The book club is designed to enhance students’ understanding of equity within the context of health research. Each cohort reads one nonfiction book that focuses on themes relevant to health equity. Discussions are primarily facilitated by CHET GRA mentors but students also have the opportunity to lead discussions based on assigned readings. These discussions encourage critical thinking about systemic inequities and justice, with students engaging in reflection activities or responding to discussion questions to prepare for each session.

#### Summer research conduct

After the didactic training and receiving IRB approval, students transition to their home communities or places of residence for the summer to pursue their research projects. SPARKlers receive a three-month stipend, recognizing that students from backgrounds traditionally underrepresented in scientific research generally cannot afford to forfeit summer income-earning potential. SPARKlers dedicate 25 hours per week to their projects during the summer which includes completing literature reviews, meeting with their CHET GRAs and research mentors, engaging in primary data collection, and participating in monthly group check-ins and professional development activities. Some students obtain support from community mentors who help them develop appropriate research questions, recruitment strategies, data collection approaches, and, eventually, dissemination of the research findings to participants.

#### Support for scholarly products and professional advancement

Consistent with SPARK’s overall goal, students learn how to develop professional presentations, expand their professional networks, and publish articles. Students work closely with their mentorship team and participate in small training sessions for support on data preparation, analysis, abstract writing, and research presentations. During the fall semester, students applying for graduate programs, jobs, fellowships, or other professional advancement opportunities may receive help drafting and reviewing application materials, letters of recommendation, and preparing results of their research project to submit as writing samples for graduate and professional school applications. This support, which includes career counseling, providing letters of reference, networking assistance, and support for publications and presentations, continues for five years after program completion. Such support not only allows us to continue to enrich the scholar but also facilitates career tracking and outcomes assessment.

## Results

### Participants

All SPARKlers are from traditionally underrepresented groups or disadvantaged backgrounds as defined by the NIH[15]. Of the 11 SPARKlers who have completed the program, 7 identify as African American or Black; 1 as Latinx, Hispanic, or Latino; and 3 identify as being from underserved rural or low-income communities.

### Program assessment

Eleven students participated in the first three SPARK cohorts (2019–2021) and have completed the program. All students provided subjective assessments of the program and their skill development via a Qualtrics© survey. The survey developed for SPARK encompassed best practices currently utilized in programmatic evaluation, tailoring efforts towards gauging increases in confidence, knowledge, and overall participant satisfaction. Open-ended items in the above student surveys as well as exit interviews conducted with students in cohorts 2 and 3 (*n* = 8) served as the basis for qualitative insights and complemented survey data. Exit interview data were not collected from cohort 1 students due to challenges faced during the onset of the COVID-19 pandemic. Where applicable, evaluation data from SPARKlers in cohort 1–3 were aggregated.

Assessments from research mentors were introduced after cohort 1 completed the program, with the research mentors having the option to complete an exit interview (cohort 2, *n* = 4 because one student had two mentors; cohort 3, *n* = 2) or a survey (cohort 3, *n* = 3). We analyzed these data with basic descriptive statistics and, in the case of the open-ended questions, qualitative template coding.

### Quantitative assessment

**Figure 1.**
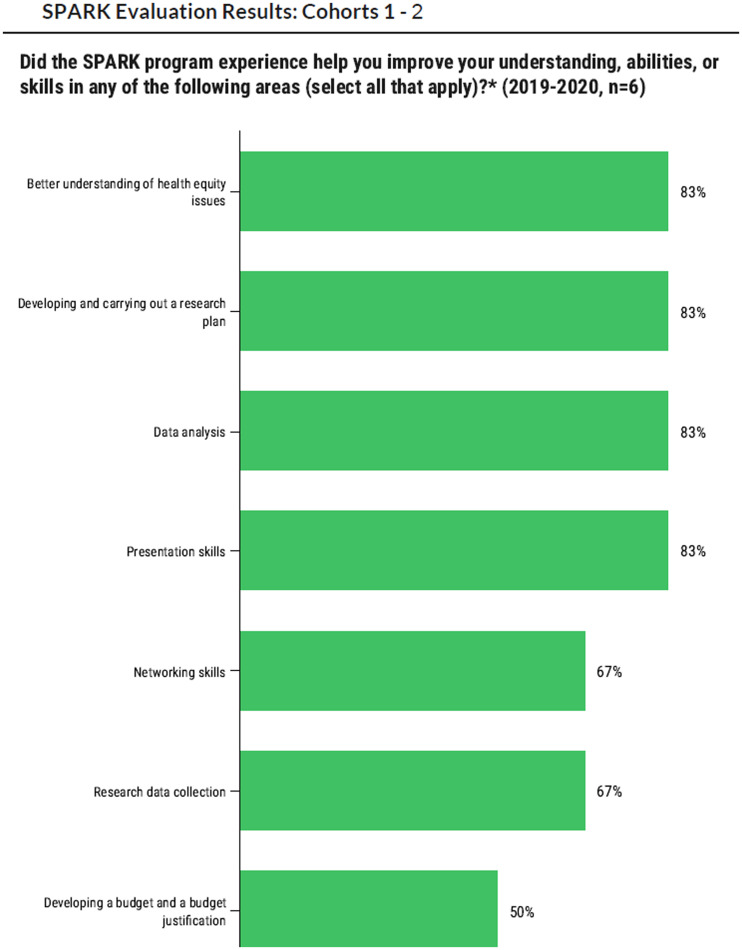
Survey assessment, knowledge and skill acquisition.

**Figure 2.**
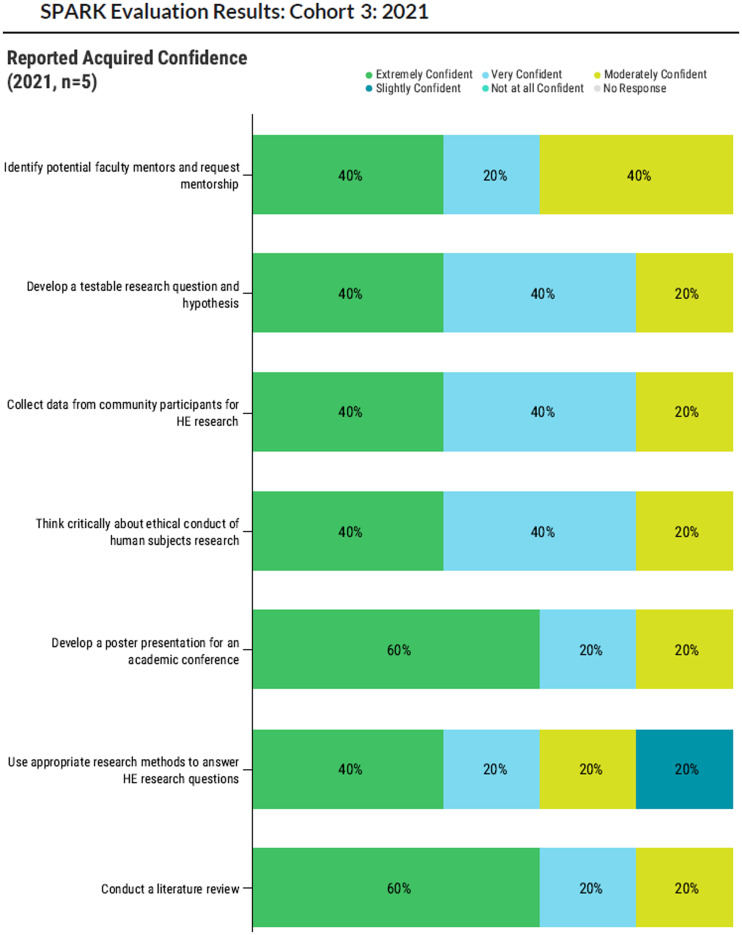
Survey assessment, change in confidence.

Figures 1 and 2 summarize survey results from cohorts 1–3 of SPARK students. Overall, all three cohorts found the SPARK training series very useful, with the one-on-one research meetings, Health Equity 101, and Research 101 sessions receiving particularly positive ratings. All students agreed that the educational training improved their understanding of health equity and that their relationship with their research mentors increased their confidence to challenge themselves to achieve new goals and explore alternatives in their research. *Skill development*: Most students (83%) indicated that they were now able to develop and carry out a health equity research plan and that SPARK improved their data analysis abilities (83%) and presentation skills (83%). *Confidence*: When asked to rate gains in confidence in key activities (not at all confident = 1 to extremely confident = 5), students reported high gains in confidence levels (extremely confident or very confident) pertaining to their ability to develop a testable research question and hypothesis (80%), use appropriate research methods to address research questions (60%), think critically about the ethical conduct of human subjects research (80%), conduct a literature review (80%), collect data from community participants (80%), as well as develop and present a poster (80%). Across all cohorts, all SPARK participants stated they would recommend the SPARK program to others.

### Qualitative assessment

Table 3 shows the major themes that emerged from qualitative data collected from students. Students highlighted that the first-hand research experience they received was unique and unavailable to their peers who did not participate in the SPARK program. Experiencing the entire life cycle of a research project as a Principal Investigator – from conducting a literature review to developing a hypothesis to the final presentation – was consistently cited as beneficial. Additional benefits of SPARK participation reported by the students include undertaking a comprehensive literature review, improving data analysis skills, expanding networking opportunities, navigating the IRB application process, and building confidence in performing independent research. SPARK participants reported that they felt supported throughout the program by both their mentors and administrative staff but also desired more time in the program to carry out their research and network.

**Table 3.** Themes from participants’ assessment of Students Participating as Ambassadors for Research in Kentucky (SPARK) Program, 2019–2021

Theme	Exemplary Quote(s)
New skill acquisition	“I knew nothing about IRB research before SPARK^†^, but that class taught me the basics. The one-on-one meetings were helpful because they gave me the opportunity to ask questions, create a timeline for my research, and work through any concerns or problems I had during the entire process.”
Confidence boost	“[SPARK] really made me feel more confident or comfortable engaging in public health literature as a whole. Additionally, my [Research] mentor was incredibly helpful in preparing me for the CCTS Conference and ensuring that everything went smoothly for me.”
Joining a community of scholars	“Amazing opportunity in terms of being able to learn from researchers, especially the CCTS Conference, have met lifelong friends. Conducting research especially human and health equity from idea, IRB, research I learned a lot.”
Enriching mentorship experience	“[Mentors] guided me through the process of independent research, IRB submission and response.”“[Research Mentor] closely related to study, made it easy to connect and recruit participants.”
Expanded professional development	“I got a research intern position on a COVID-19 Moderna study that focused on college campuses. This even led me to meet and ask a question to Dr Anthony Fauci. None of that would have happened without SPARK.”“Surprised about how much I enjoyed research!”
Time constraints	“The short amount of time allotted to develop a research question, conduct the study and analyze data made things difficult and stressful at times; however, I was still able to learn a lot and gained valuable research experience.”“Wished we would have had more time with everyone, like luncheon, felt rushed. More time to develop community and relationship building events.”

Beginning in cohort 2, research mentors provided feedback about the SPARK program and their overall experience serving as mentors. All research mentors (*n* = 9) reported being intensively and directly involved in SPARKlers’ research projects and supporting their students’ academic and career goals. While some research mentors noted challenges, such as students’ inability to consistently meet deadlines (*n* = 3), demonstrate effective communication (*n* = 3), or sustain motivation (*n* = 2), they all believed the program was an overall benefit to students. Some research mentors also identified managing mentor relationships, working through the research process, and analyzing data as specific skills they transferred to students. All research mentors noted that the program was beneficial as it allowed students to undertake the entire research process as a Principal Investigator. Several (*n* = 4) felt that completing an IRB application was rewarding for students although one research mentor stated that undergoing the IRB process was too time-consuming, causing delays to the students’ research project. Several mentors (*n* = 5) also reported that the mentoring process benefited them in unanticipated ways, including greater immersion in a health equity research center, professional and personal growth as a mentor, and the satisfaction of being an advocate for the students.

### Professional development outcomes

Eleven students have completed SPARK, with three currently finishing their undergraduate degree requirements and applying to professional schools (Table 4). Of the eight alumni who have completed their undergraduate degrees, six are currently enrolled in graduate school. The remaining two students are preparing to apply for graduate school. Additionally, three of the eight program alumni began professional careers focused on health equity in clinical research and administration before pursuing graduate studies or while earning their graduate degrees.

**Table 4.** Current placement of Students Participating as Ambassadors for Research in Kentucky (SPARK) students

	Major(s) when enrolled	Current Placement/Status
1	Psychology	PhD Student, Human Development and Family Studies.
2	Medical Laboratory Science	MSc Student, Biomedical Sciences.
3	Public Health	MSc Student, Medical Science, Clinical Research Associate
4	Human Health Sciences	MSc Student, Genetic Counseling
5	Neuroscience	MSc Student, Medical Science.
6	Psychology	Research Coordinator
7	Biology and Neuroscience	Junior, University of Kentucky.
8	Public Health	Senior, University of Kentucky.
9	Biology and Mathematics	Senior, University of Kentucky.
10	Public Health and Environmental Sustainability	MA Student, Geography, Teaching Assistant.
11	Biology	Post Bac Student, Neuroscience Research Education.

## Discussion

This article describes the SPARK program, a comprehensive experience designed to introduce undergraduate students from underrepresented and disadvantaged backgrounds and communities to health equity research. SPARKlers indicate high cohort satisfaction with the didactic sessions and the mentor meetings. SPARK also produced improvements in skill acquisition, networking opportunities, and supportive mentorship, with participants expressing a willingness to recommend the program to others.

Over these three cohorts, we have learned several important lessons. First, while SPARK evaluations demonstrate a high level of program acceptability, like all programs, ongoing process improvements are needed. All participants noted the need for improved communication on the part of students, mentors, and program leadership. Students requested additional time to connect with one another in the Fall semester in Year 1 of the program, and the SPARK Executive Committee identified a need for better communication with research mentors and program leadership. These observations and feedback led to changes in the program timeline that increased interaction while maintaining the research project timeline. Changes included in-person didactic training sessions and professional development activities, earlier research mentor identification, enhanced orientations for students and research mentors, monthly check-in messages from the CHET faculty mentor to individual research mentors, and additional in-person professional development activities scheduled for the Spring Year 2 semester. Additionally, the SPARK Executive Committee began meeting biweekly to identify challenges earlier and address them appropriately.

Second, students have encountered challenges in receiving IRB approval in a timely manner throughout the program. This has caused significant shifts in research timelines and expectations for project completion. To help overcome this challenge, the Executive Committee restructured training on this topic and engaged the UK Office of Research Integrity, which governs the IRB process, to assist students in developing and completing their applications. Despite timeline challenges, students have found the process of experiencing the life cycle of a research project rewarding.

Third, the success of the SPARK program depends on the financial support of numerous partners to cover participant costs, graduate students, and general operating expenses. SPARK students each receive $5,000 in summer stipends and $3,000 in research project funds. The program does not provide housing or tuition costs for SPARKlers, but mileage reimbursement is covered as a research expense for students required to travel for their project implementation. Faculty mentors and community mentors receive $1,000 stipends for their efforts. Beginning with the Cohort 3 program expansion, the SPARK Program covers tuition, a 12-month stipend, and health insurance for two doctoral-level graduate students to provide one-on-one mentorship to SPARKlers. Additional costs include funding for professional development activities, experiential site visits, and travel and publication costs for students to disseminate their findings in peer-reviewed journals and at external conferences. The total 16-month intensive and individualized health equity research experience per-student cost for the program is $20,000. Obtaining internal funds is challenging due to other undergraduate research programs on campus, the level of investment required to support the robust training, mentoring, and professional development structure, and the diverse academic and research interests of students that are not confined to one department or unit. Some contributions directly cover expenses, while others defray program costs through in-kind donations of time and expertise. SPARKlers’ research mentors receive modest stipends but largely are rewarded for their mentorship and expertise through goodwill. This same sense of purpose and mission pertains to members of the SPARK Executive Committee, who donate their time. This financial challenge impacts the faculty and staff support of the program, which could be supported by NIH extramural funding to increase the number of individuals from underrepresented and disadvantaged backgrounds in health research and the workforce.

Finally, fulfilling the program mission involves extensive mentorship and training since students enter the program with limited research experience. This training orientation requires consistent hands-on guidance to ensure the success of a small number of students at a time. Existing research confirms this orientation and demonstrates, for example, that smaller class sizes are associated with greater likelihood of receiving an A in a course [18]. In order to support student programmatic and career success, a significant investment of time is required to build trusting relationships and guide students in their independent research. SPARK Program participants are ambitious undergraduates who face unique challenges as students from traditionally underrepresented and disadvantaged backgrounds in academia [2]. Meeting these students where they are while accommodating their course schedules, extracurricular activities, and pursuit of graduate school, professional school, and workforce positions is labor intensive. Each member of the SPARK mentorship team plays a role in supporting students that often extends beyond their program deliverables. This support is both crucial to student success and at times challenging for mentors to provide.

While demonstrating promise (for example, nearly all SPARK alumni are enrolled in graduate programs), evaluation of the SPARK program is made challenging by several factors. The modest cohort size makes traditional quantitative evaluation less feasible; the unusual circumstances of conducting community research during a pandemic defy establishing regularity of program practices; and our attempt to employ consistent quality improvement has led us to enact programmatic changes, which also complicate a standard evaluation. Examining data from subsequent cohorts and longer-term follow-up is necessary to determine impact on contributing to the next generation of health equity scholars.

### Future steps

Students from cohort 4 (2022, *N* = 6) will complete the program in May 2024 and cohort 5 (2023, *N* = 6) just began their Spring Year 1 didactic coursework.

The Executive Committee is in the process of having the SPARK curriculum approved as an official UK course eligible for registration with course credit by all students in the program. Additional future steps include increasing the number of students that submit manuscripts and present at external conferences, engaging in long-term annual evaluation of all program alumni, expanding the program beyond six students per cohort, expanding the program to additional academic institutions, including historically Black colleges and universities, and strengthening relationships with health-related research programs at both ends of the training continuum to enhance recruitment and retention in health equity research careers.

## Conclusion

The SPARK Program offers an intense, individualized pathway to develop the next generation of health equity scholars. An increasingly diverse world with persistent and emerging health challenges calls for an equally diverse workforce with the capacity to implement transformative solutions. The SPARK Program provides a unique experience for underrepresented and disadvantaged undergraduate students because of its focus on health equity research rather than laboratory research, its length and comprehensiveness of training, and its focus on undergraduates from traditionally underrepresented and disadvantaged backgrounds. Many undergraduate research programs provide students with only summer mentorship, and more comprehensive programs predominantly focus on laboratory and basic science research [10,13,14]. Well-developed research mentorship programs for traditionally underrepresented and disadvantaged undergraduate students that provide training in health equity research are effective in growing and diversifying the workforce needed to improve health outcomes for all, and serve as a worthy investment for those committed to health equity.

## Supporting information

Arthur et al. supplementary material Arthur et al. supplementary material

## Supplementary material

The supplementary material for this article can be found at https://doi.org/10.1017/cts.2024.688.
