# Supplementary figures and images for: Students Participating as Ambassadors for Research in Kentucky (SPARK): A health equity undergraduate research training program
Source: J Clin Transl Sci. 2024 Dec 26;9(1):e21. doi: 10.1017/cts.2024.688 (PMC11795858; doi:10.1017/cts.2024.688)

Supplementary Materials - Syllabus for Didactic Training
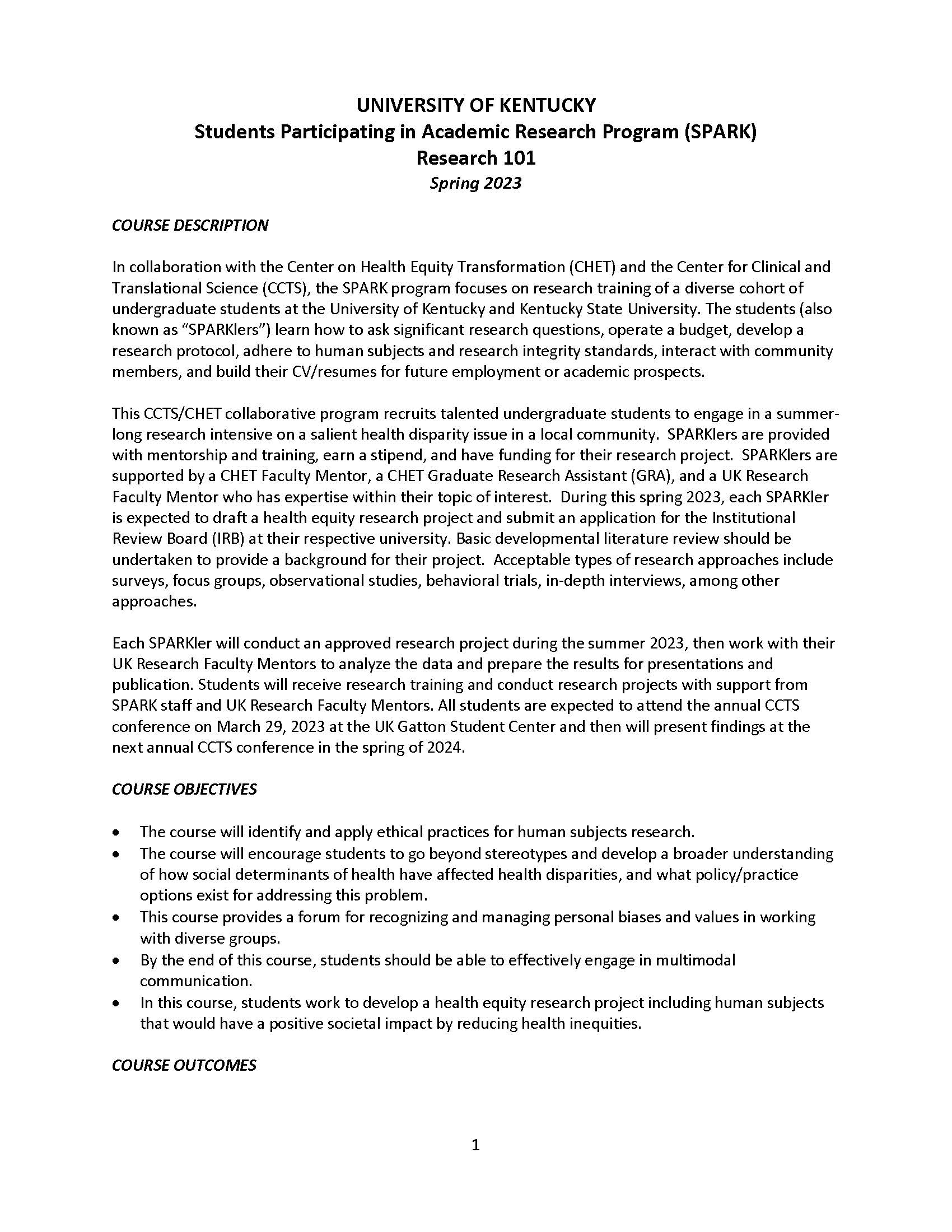


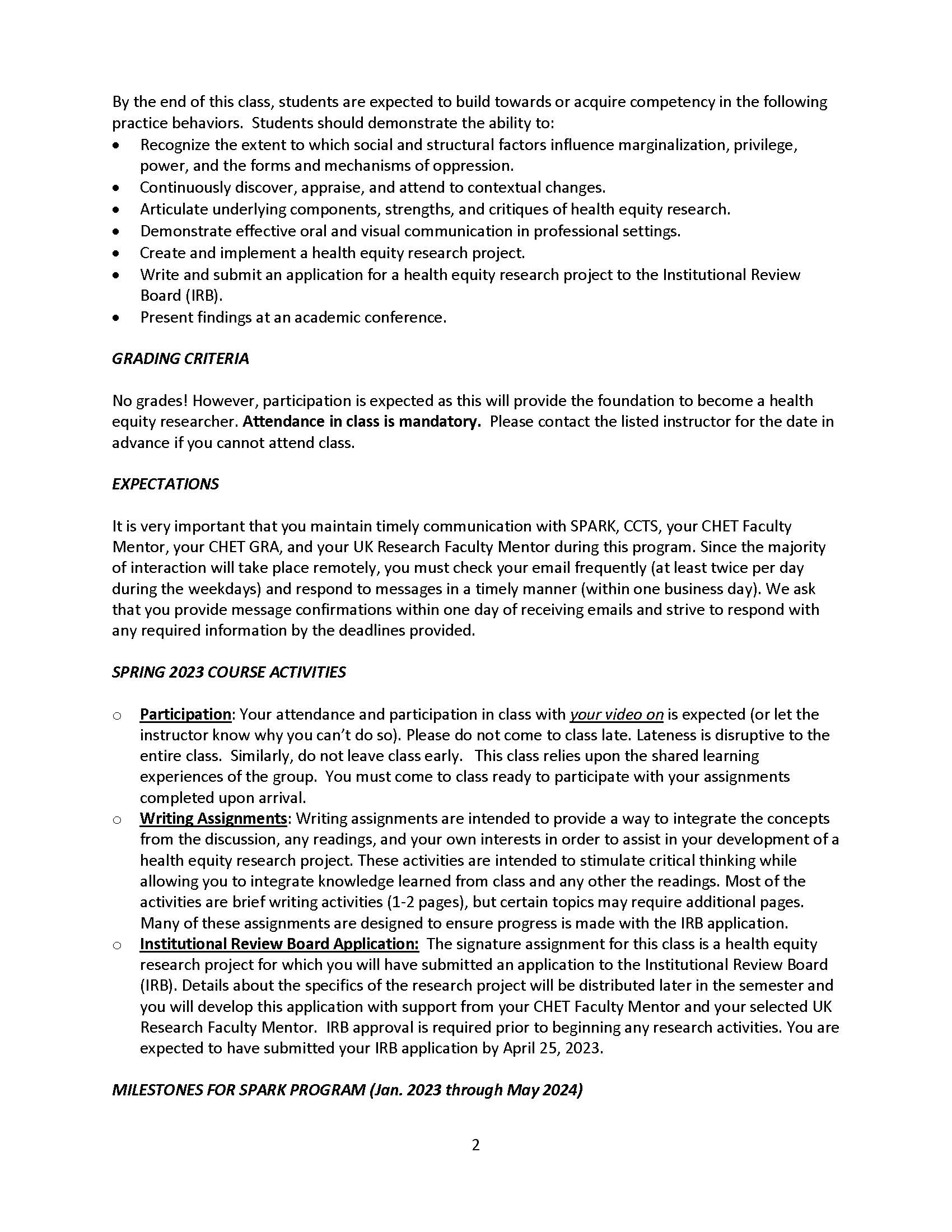

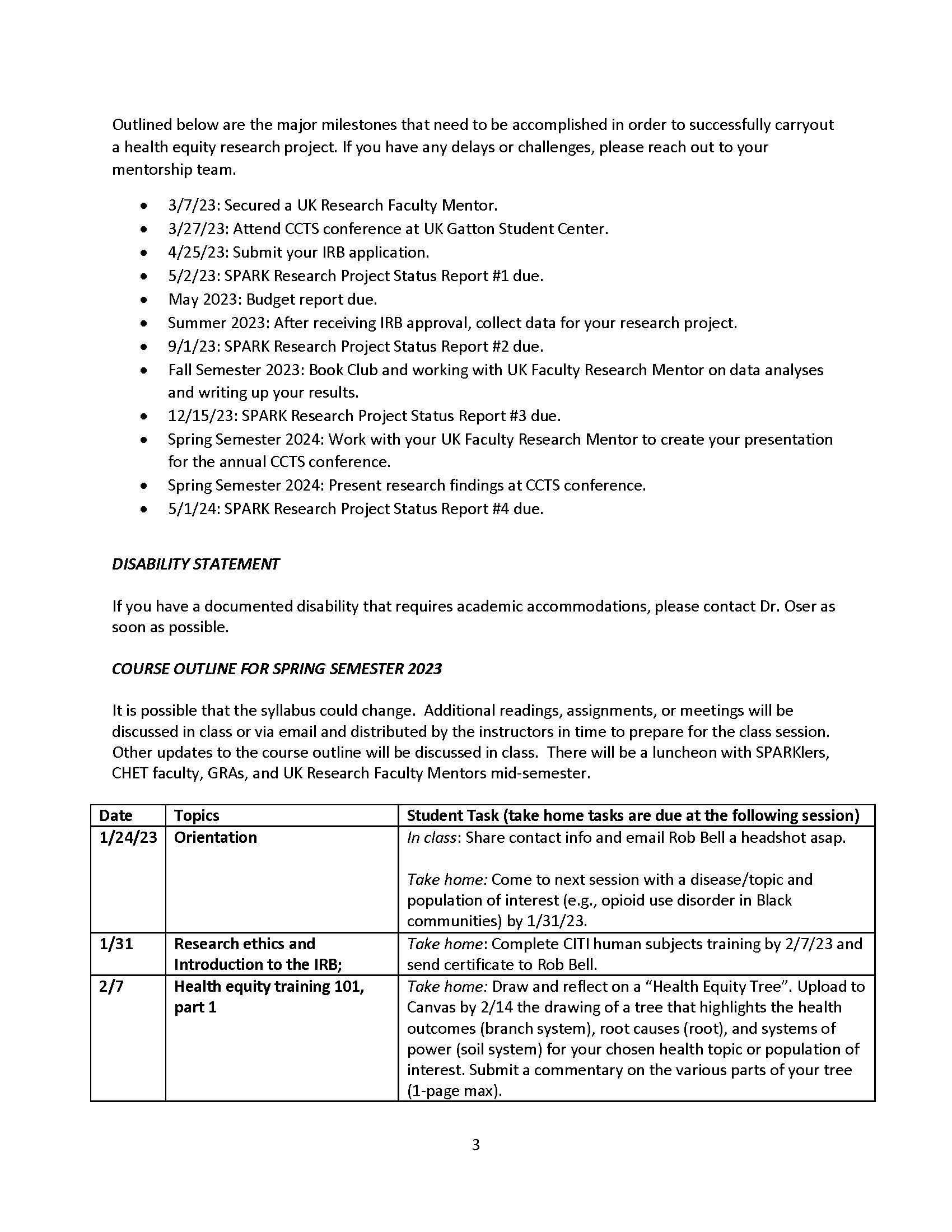

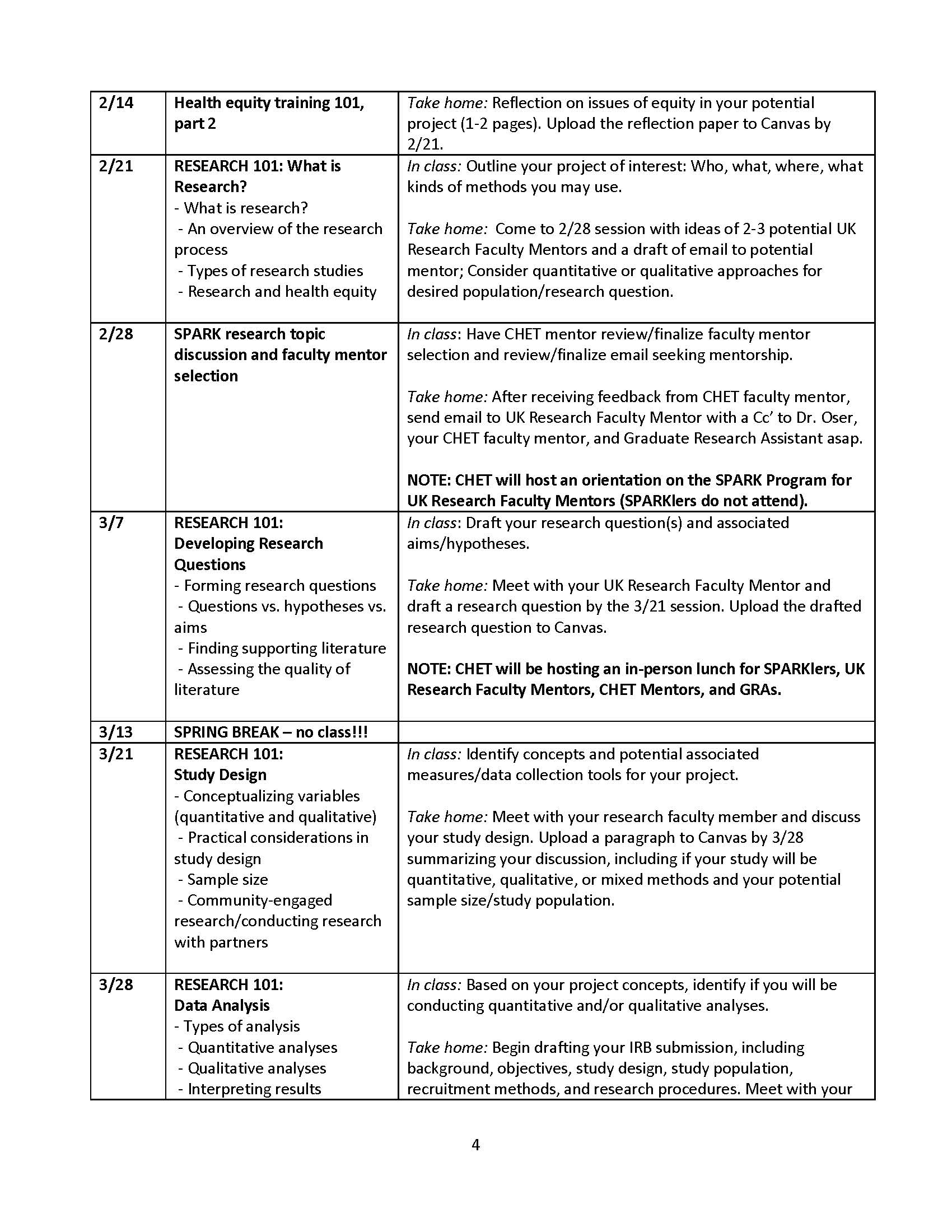

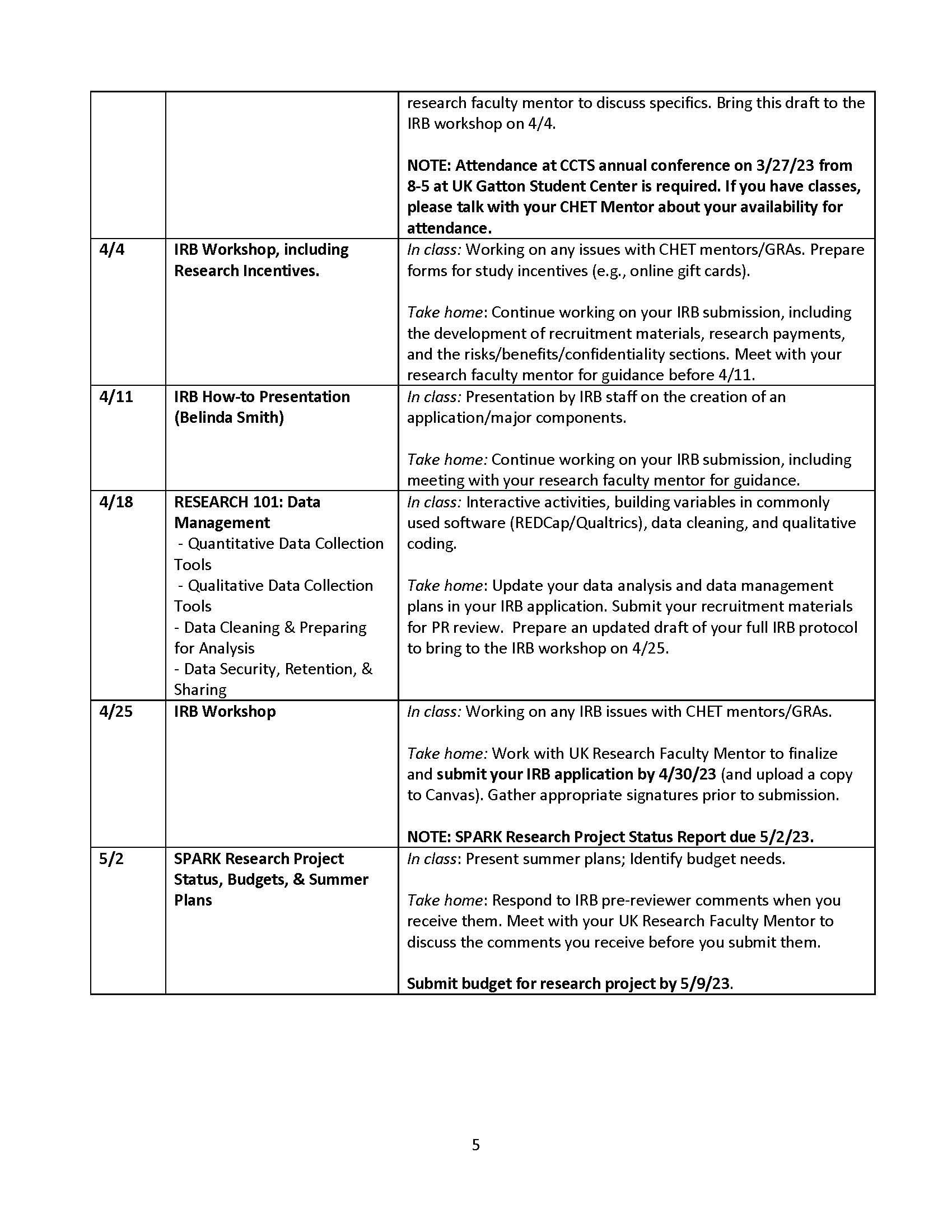

Supplement: Arthur et al. supplementary material [file S2059866124006885sup001.docx]
